# *WUSCHEL-RELATED HOMEOBOX 2* is important for protoderm and suspensor development in the gymnosperm Norway spruce

**DOI:** 10.1186/s12870-016-0706-7

**Published:** 2016-01-19

**Authors:** Tianqing Zhu, Panagiotis N. Moschou, José M. Alvarez, Joel J. Sohlberg, Sara von Arnold

**Affiliations:** Department of Plant Biology, Uppsala BioCenter, Swedish University of Agricultural Sciences and Linnean Center for Plant Biology, PO-Box 7080, SE-75007 Uppsala, Sweden

**Keywords:** Norway spruce, Protoderm, Somatic embryo, *WUSCHEL-RELATED HOMEOBOX 2*

## Abstract

**Background:**

Distinct expression domains of *WUSCHEL-RELATED HOMEOBOX* (*WOX*) gene family members are involved in patterning and morphogenesis of the early embryo in Arabidopsis. However, the role of *WOX* genes in other taxa, including gymnosperms, remains elusive. Here, we use somatic embryos and reverse genetics for studying expression and function of *PaWOX2*, the corresponding homolog of *AtWOX2* in the gymnosperm *Picea abies* (*Pa*; Norway spruce).

**Results:**

The mRNA level of *PaWOX2* was transiently up-regulated during early and late embryogeny. *PaWOX2* mRNA in early and early late embryos was detected both in the embryonal mass and in the upper part of the suspensor. Down-regulation of *PaWOX2* during development of early embryos resulted in aberrant early embryos, which failed to form a proper protoderm. Cells on the surface layer of the embryonal mass became vacuolated, and new embryogenic tissue differentiated from the embryonal mass. In addition, the aberrant early embryos lacked a distinct border between the embryonal mass, and the suspensor and the length of the suspensor cells was reduced. Down-regulation of *PaWOX2* in the beginning of embryo development, before late embryos were formed, caused a significant decrease in the yield of mature embryos. On the contrary, down-regulation of *PaWOX2* after late embryos were formed had no effect on further embryo development and maturation.

**Conclusions:**

Our data suggest an evolutionarily conserved function of *WOX2* in protoderm formation early during embryo development among seed plants. In addition, *PaWOX2* might exert a unique function in suspensor expansion in gymnosperms.

**Electronic supplementary material:**

The online version of this article (doi:10.1186/s12870-016-0706-7) contains supplementary material, which is available to authorized users.

## Background

The basic plant body pattern is set up during embryogenesis. In seed plants, this body plan has been described as the superimposition of two patterns: an apical-basal and a radial pattern [1]. The molecular processes that establish this primary body plan have mainly been studied in the angiosperm model species Arabidopsis (*Arabidopsis thaliana*). In contrast, knowledge about the molecular regulation of embryo development in gymnosperms is limited, partly owing to the lack of identified zygotic embryo defective mutants and genetic tractability. However, by using somatic embryos and reverse genetics it has been possible to study the regulation of embryo development in conifers [2]. We are studying the early stages of embryo development in Norway spruce and especially the role of members of the *WUSCHEL-RELATED HOMEOBOX* (*WOX*) gene family, which encode transcription factors that play important roles in the determination of cell fate during embryogenesis in angiosperms [3, 4].

Angiosperms and gymnosperms separated approximately 300 million years ago and expectedly their patterning during embryogenesis differs significantly. For convenience, embryogenesis in Arabidopsis can be divided into three general phases, described as proembryogeny, early embryogeny (globular-stage to heart-stage transition) and late embryogeny [5]. Proembryogeny begins after fertilization. An asymmetric cell division of the zygote generates a smaller apical cell and a larger basal cell. The apical cell is the founder of the embryo proper, while the basal cell develops into the suspensor. The apical cell undergoes several rounds of stereotyped asymmetric divisions, giving rise to cells with positionally determined cell fate. At the beginning of early embryogeny, in the 8-cell embryo proper, a single round of tangential divisions separate the outer layer of eight cells from the eight inner cells [6]. The inner cells are founder cells of the ground tissue and vascular elements. The outer cells form the protoderm which will become the epidermis. Plant epidermis is characterized by the secretion of lipids and waxes to its outer cell wall [7]. The continuous hydrophobic layer forms the cuticle. Owing to the periclinal cell division pattern in the protoderm, the protodermal cells remain essentially separated from the inner cells throughout embryogenesis. Root and shoot meristems are established during the transition from globular to heart-stage. After the heart-stage the suspensor is degraded by programmed cell death [5]. During late embryogeny, there is a switch from pattern formation to storage product accumulation.

In conifers, the sequence of embryo development can also be described by three phases: proembryogeny, early embryogeny, and late embryogeny [8]. Proembryogeny begins when the zygote undergoes several rounds of nuclear duplications. The produced free nuclei are first arranged in a tier before cellularization. After a cell division, two tiers are formed. The cells in the upper tier elongate to form a functional suspensor and the cells in the lower tier divide creating the embryonal mass (analogous to the embryo proper in angiosperms). Early embryogeny begins with the elongation of the embryonal suspensor. Cells in the outer layer of the embryonal mass divide mainly anticlinally, but also periclinally giving rise to additional internal layers [8], unlike angiosperms where only anticlinal cell divisions take place. Nevertheless, the outer cell layer in the embryonal mass in conifer embryos defines a functional protoderm [9, 10]. Late embryogeny is a period of histogenesis and organogenesis. Early during this phase, the suspensor cells are dismantled by programmed cell death [11], and the root and shoot apical meristems are delineated.

Members of the *WOX* gene family play important roles in determining cell fate during plant development. Phylogenetic analyses have identified three major clades in the *WOX* gene family: the modern (*WUS* and *WOX1-7*), the intermediate (*WOX8*, *9*, *11*, and *12*) and the ancient clade (*WOX10*, *13*, and *14*) [12]. In the gymnosperm Norway spruce (*Picea abies*), 11 *WOX* genes that belong to all major clades, have been identified [13].

All *WOX* genes examined show very specific expression patterns, both spatially and temporally, which are important for their molecular functions [14]. *WOX2*, *WOX8* and *WOX9* have been implicated in patterning and morphogenesis of the early embryo in Arabidopsis [3, 15, 16]. *AtWOX2* is expressed in the egg cell and the zygote [3]. After the first cell division, *AtWOX2* marks the apical cell, while, *AtWOX8* and *AtWOX9* mark the basal cell. *AtWOX8* and *AtWOX9* share redundant functions [16]. The Arabidopsis *wox8wox9* double mutant shows aberrant orientation of cell division planes in the embryo [16]. Previously, we showed that the Norway spruce gene *PaWOX8/9* is the orthologue of *AtWOX8* and *AtWOX9* [17]. Similarly to Arabidopsis, down-regulation of *PaWOX8/9* causes disturbed orientation of the cell division plane and cell fate determination during early embryo pattern formation suggesting that *PaWOX8/9* exerts an evolutionarily conserved function.

It has previously been shown that *PaWOX2*, which is highly similar in sequence to *AtWOX2*, is expressed during embryo development in gymnosperms [13, 18–20]. Herein we report that *PaWOX2* is transiently expressed in the embryonal mass and in the upper part of the suspensor in early and early late embryos. Furthermore, by using reverse genetics, we functionally characterize *PaWOX2*. Down-regulation of *PaWOX2* during development of early embryos results in aberrant early embryos, which lack distinct embryonal mass and suspensor domains, a proper protoderm, and have shorter suspensor cells. This suggests an evolutionarily conserved function of *WOX2* in protoderm formation early during embryo development among seed plants. In addition, *PaWOX2* might exert a unique function in proper suspensor cell expansion in gymnosperms.

## Materials and methods

### Plant material

The embryogenic cell line 61:21 of Norway spruce (*Picea abies* L. Karst) has been used in this study. The cell line was established as described by Högberg et al. [21]. The cell line was stored in liquid nitrogen and thawed a couple of months before the start of experiments. After thawing the cell cultures were maintained as described previously [2]. Briefly, proembryogenic masses (PEMs) were maintained on solidified proliferation medium containing the plant growth regulators (PGRs) auxin and cytokinin. To stimulate development of somatic embryos the cultures were first transferred to pre-maturation medium lacking PGRs for one week and then to maturation medium containing 30 μM abscisic acid (ABA). Early embryos (EEs) differentiated after one week on maturation medium; early late embryos (LE1s) and LE2s developed after two and three weeks on maturation medium, respectively; maturing embryos (ME1s), characterized by the initiation of cotyledons, developed after five weeks on maturation medium. ME2s (almost fully matured embryos) and ME3s (fully matured embryos) developed after about eight weeks on maturation medium.

### RNA extraction, cDNA synthesis and quantitative real-time PCR

Samples for analyzing the mRNA level of *PaWOX2* (accession number: AM286747) during embryo development, were collected from nine sequential developmental stages: PEM1 (after seven days on proliferation medium), PEM2 and PEM3 (after three and seven days on pre-maturation medium respectively), EE, LE1, LE2, ME1, ME2 and ME3 (after one to eight weeks on maturation medium). The sampling was performed at mid-day and samples were frozen in liquid nitrogen and stored at −80 °C after collection.

Total RNA was isolated using the Spectrum Plant Total RNA kit (Sigma-Aldrich, USA) according to the manufacturer’s instructions. For each sample, 1 μg of total RNA was reverse transcribed with RevertAid H Minus First Strand cDNA Synthesis Kit (Fermentas, Thermo Scientific, Sweden) using an equimolar ratio of random and oligo-dT primers according to the manufacturer’s instructions.

Quantitative real-time PCR (qRT-PCR) was performed as described previously [17]. Three reference genes, *CELL DIVISION CONTROL2* (*PaCDC2*)*, ELONGATION FACTOR 1* (*PaEF1*) and *PHOSPHOGLUCOMUTASE* (*PaPHOS*) were used [22]. Two to three biological replicates, each with three technical replicates were performed for each test. The primer sequences are presented in Additional file 1: Table S1. Statistical analysis was done by *t*-test.

### RNA in situ hybridization

For RNA *in situ* hybridization (ISH) the following materials were used: ovules from cones collected in the end of June and somatic embryos (EEs, LE1s and ME1s). The ovules were fixed and embedded as described by Karlgren, et al. [23]. The somatic embryos were fixed in 3.7 % formaldehyde, 5.0 % acetic acid and 50 % ethanol overnight and embedded in Technovit 8100. A gene-specific fragment was used as a probe. The probes were prepared with DIG RNA Labeling Kit (see primer sequences in Additional file 1: Table S1) (Sigma-Aldrich, USA). *In situ* hybridization was performed essentially as described by Karlgren et al. [23]. Sections of 10 μm were hybridized to digoxigenin-labeled RNA probes. The pictures were processed using Adobe Photoshop CS6 13.0 software.

### RNA interference vector construction

The coding sequence (CDS) of *PaWOX2* was amplified from a cDNA library of early somatic embryos of Norway spruce [13]. The full-length CDS was subcloned into the pJET1.2/blunt cloning vector using the CloneJET™ PCR Cloning Kit (Fermentas, Thermo Scientific, Sweden). To obtain RNA interference (RNAi) constructs, two overlapping fragments of *PaWOX2* were amplified and fused to form a hairpin structure for *PaWOX2* (Additional file 2: Figure S1). To fuse these fragments, EcoRI and BamHI digestion sites were added on forward primers as linkers. The hairpin was confirmed by sequencing. Primers are presented in Additional file 3: Table S2.

Hairpin structures were introduced into pENTR™/D-TOPO® (Invitrogen, Carlsbad, CA, USA) and then inserted by *att* site LR recombination into the destination vector pMDC7 [LexA-VP16-ER (XVE) *β*-estradiol inducible promoter, which is derived from the pER8 vector and contains the estrogen receptor-based transactivator XVE] [24, 25] or pMDC32 (35S constitutive promoter) [26]. Hairpin structures were confirmed by sequencing. Vectors were introduced by electroporation into *Agrobacterium tumefaciens* strain GV3101.

### Transgenic cell lines

Embryogenic cultures were transformed by co-cultivation with *A. tumefaciens* as described previously [17]. Stably transformed lines were selected after four weeks. Genomic DNA was isolated from PEMs from selected lines by using the DNeasy plant mini kit (Qiagen, Germany), according to the manufacturer’s instructions. Transformed lines were confirmed by PCR.

The mRNA level of *PaWOX2* in *PaWOX2* RNAi lines was analyzed by qRT-PCR. In the case of the inducible XVE-*WOX2i* lines, cultures were induced with *β*-estradiol (10 μM) for 48 h before the analysis. The lines 35S:*WOX2i.2*, 35S:*WOX2i.3*, 35S:*WOX2i.4* and XVE-*WOX2i.12* were selected for further studies (Additional file 4: Figure S2). The untransformed 61:21 line was used as a control. In addition, in the time-laps tracking experiments we also included a transformed control line (T-control), expressing the reporter GUS (*β*-glucuronidase) under the 35S promoter.

To study if *PaWOX2* regulates cell division, the mRNA level of nine cell-cycle-regulating genes [*PaRETINOBLASTOMA-RELATED PROTEIN-LIKE* (*PaRBRL*)*, PaEXTRA SPINDLE POLES* (*PaESP*), two *E2F* family genes (*PaE2FABL*) and five *CYCLIN-LIKE* (*PaCYCLs*) genes] were analysed in EEs from the control and line 35S:*WOX2i.4* by qRT-PCR as previously described [17].

### Morphological analysis

Samples of EEs for morphological analysis were collected from the control and lines 35S:*WOX2i.2*, 35S:*WOX2i.3*, 35S:*WOX2i.4* and XVE-*WOX2i.12* after one week on maturation medium. The samples were embedded by mixing with 2 ml of 1.2 % (w/v) Seaplaque agarose (FMC BioProducts, USA) in 60 mm Petri dishes. The length and width of suspensor cells of about 40 EEs from the control and 35S:*WOX2i* lines were measured using the ImageJ software (ver. 1.48 g) [27].

To further study embryo morphology, 26 LE1s from the control and line 35S:*WOX2i.4* were scanned with a Zeiss 780 confocal microscope (Carl Zeiss AG), using the 488 nm Argon laser line, and the 20x objective (NA = 0.80).

For histological analysis, EEs from the control and line 35S:*WOX2i.4* were fixed in 3.7 % formaldehyde, 5.0 % acetic acid and 50 % ethanol overnight. Subsequently, samples were dehydrated in 50, 75, 90 and 100 % ethanol series. Finally, the samples were embedded in Technovit 8100 (Kulzer, Wehrheim, Germany). The embryos were processed for serial sectioning (10 μM) on a Zeiss HM 355 microtome.

The cuticle of untreated LE1s from the control and line 35S:*WOX2i.4* was stained in freshly prepared Oil Red, 0.2 % (w/v) in water, for 5 min and then washed in water [28]. The stained embryos were hand-sectioned and examined under a Zeiss Axioplan microscope in dark field with a 5x objective (NA = 0.12).

Time-lapse tracking analysis was performed to examine in great detail the developmental pattern from EE to ME. EEs from the controls (both untransformed and T-control) and lines 35S:*WOX2i.2*, 35S:*WOX2i.3* and 35S:*WOX2i.4* (50 embryos per line) were sampled after one week on maturation medium and transferred to fresh maturation medium. Embryo morphology was examined every second day for 15 days.

To study the effect of *PaWOX2* on the maturation process, after one week on pre-maturation medium cultures from the control and lines 35S:*WOX2i.2*, 35S:*WOX2i.3*, 35S:*WOX2i.4* and XVE-*WOX2i.12* were re-suspended in liquid pre-maturation medium and plated out as a thin layer on filter paper placed on maturation medium. For the XVE-*WOX2i* line, *β*-estradiol (10 μM) was added to the maturation medium, either from the start or after two weeks on maturation medium, when LE2s had already developed. The development of embryos was recorded for 14 days on maturation medium. The time points examined were 1, 3, 6, 10 and 14 days. The number of ME3s developed per initial gram of tissue was estimated after seven weeks on maturation medium.

## Results

### Expression of PaWOX2 during embryo development

**Fig. 1:**
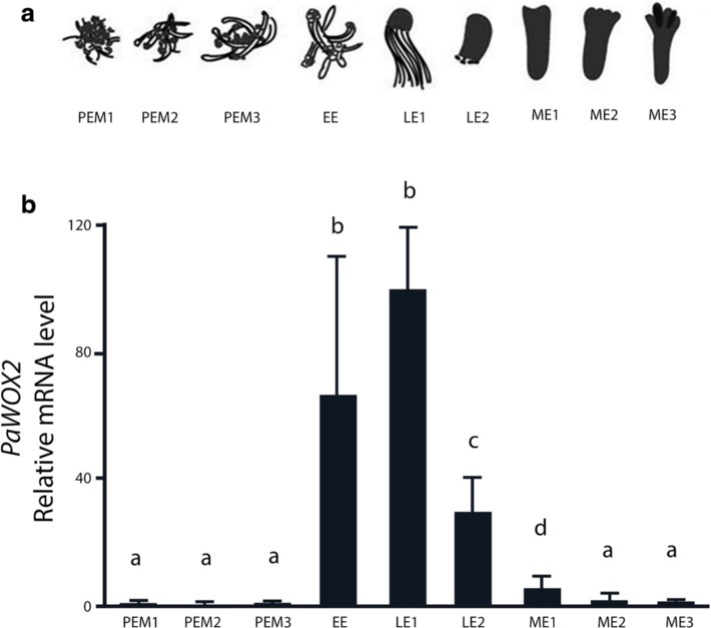
Relative mRNA level of *PaWOX2*. **a** Schematic representation of the developmental stages of embryo development. Proliferating proembryogenic masses (PEMs): PEM1, seven days after subculture to fresh proliferation medium in the presence of plant growth regulators (PGRs), PEM2 and PEM3, three and seven days after transfer to pre-maturation medium lacking PGRs; early embryo (EE), after one week on maturation medium; early late embryo (LE1) and late embryo before the formation of cotyledons (LE2) after one and two weeks on maturation medium, respectively; maturing embryo (ME1), characterized by the initiation of cotyledons after five weeks on maturation medium; ME2 (almost fully maturated embryo) and ME3 (fully maturated embryo) after about eight weeks on maturation medium. The majority of the embryos in each sample represented the designated specific developmental stage. However, since the development was not strictly synchronized, embryos from previous and subsequent stages could also be represented. **b** qRT-PCR analysis of the relative mRNA level of *PaWOX2* in nine sequential developmental stages of embryo development. The mRNA level is relative to the mRNA level of *PaWOX2* in LE1 and is normalized against three reference genes: *CELL DIVISION CONTROL 2* (*PaCDC2*)*, ELONGATION FACTOR-1* (*PaEF1*) and *PHOSPHOGLUCOMUTASE* (*PaPHOS*). The mRNA level is the mean ± SE of three biological replicates. Different letters indicate significant differences in the mRNA levels among developmental stages (ANOVA, *p* < 0.05)

It has been shown that *PaWOX2* is specifically expressed in early somatic embryos of Norway spruce [13, 19]. In order to get a higher resolution of the fluctuations of the mRNA level of *PaWOX2* during embryo development, samples from nine sequential developmental stages, spanning all three phases of embryo development, were collected for qRT-PCR analysis (Fig. 1a). The mRNA level of *PaWOX2* was low in PEMs and increased sharply upon formation of EEs (Fig. 1b). The highest mRNA level of *PaWOX2* was observed in LE1s (Fig. 1b). Thereafter, it decreased, to become almost undetectable in MEs (Fig. 1b).

**Fig. 2:**
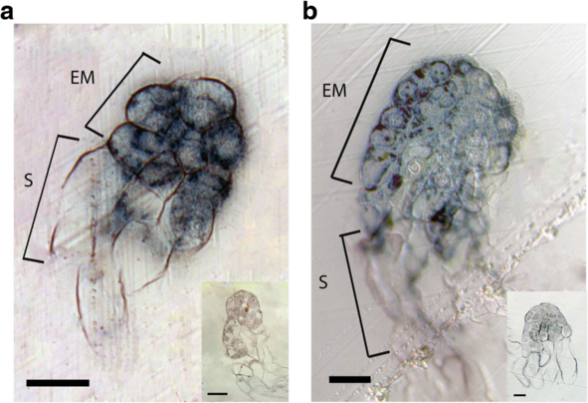
Expression pattern of *PaWOX2* in early (EE) and early late (LE1) embryos according to mRNA *in situ* localization. Hybridization signals appear as dark blue in bright field microscopy. **a** Longitudinal section of an EE. **b** Longitudinal section of a LE1. Note that signals are detected in the embryonal mass and in the upper part of the suspensor in both EE and LE1. The insert presents the sense probe control. No signal was detected after hybridization with sense probe (negative control). EM, embryonal mass; S, suspensor. Bar = 50 μm

To gain more insight into the spatial expression of *PaWOX2 in situ* mRNA hybridization was conducted on somatic embryos. *PaWOX2* mRNA was detected in the embryonal mass and in the upper part of the suspensor in both EEs and LE1s (Fig. 2). No signal could be detected in MEs (data not shown). *In situ* mRNA localization analyses were also conducted on ovules collected in the end of June at the time when zygotic late and maturing embryos had developed. Hybridization signals were detected in late embryos (Additional file 5: Figure S3A), but not in maturing embryos (Additional file 5: Figure S3C). In addition, signals were detected in the mega gametophyte residing in the front of the growing embryo (Additional file 5: Figure S3).

### PaWOX2 is required for proper protoderm formation

In order to examine the function of *PaWOX2*, we constructed stably transformed *PaWOX2* RNAi lines, using constitutive (35S; referred as 35S:*WOX2i*) or inducible (XVE; referred as XVE-*WOX2i*) promoters to drive expression of a hairpin RNA used to promote *PaWOX2* specific RNAi. The down-regulation of *PaWOX2* was confirmed by qRT-PCR (Additional file 4: Figure S2). The transcript level of *PaWOX2* in non-induced tissue was lower than in the control. In accordance, it was previously shown that the XVE promoter is partially active in Norway spruce even in the absence of *β*-estradiol [18].

**Fig. 3:**
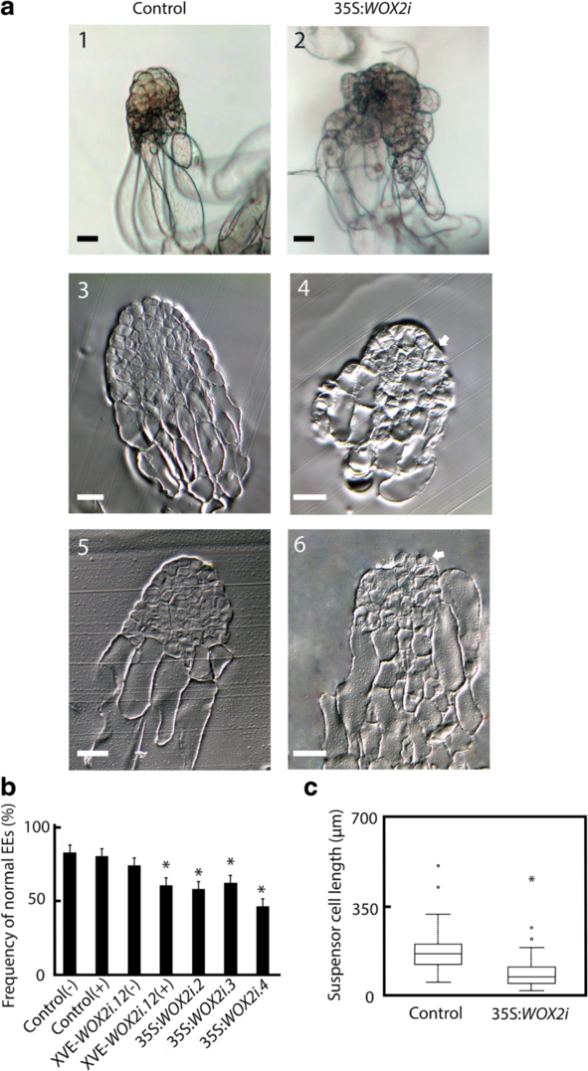
*PaWOX2* is important for the development of normal early embryos (EEs). EEs were sampled from the control and lines XVE-*WOX2i.12*, 35S:*WOX2i.2, 35S:WOX2i.3* and35S:*WOX2i.4* after one week on maturation medium. **a** A.1, Normal EE from the control. Note the smooth surface of the embryonal mass and the distinct border between the embryonal mass and the suspensor. A.2, Aberrant EE from line 35S:*WOX2i.4*. Note the vacuolated cells in the outer cell layer of the embryonal mass and the lack of a distinct border between the embryonal mass and the suspensor. A.3, longitudinal section of a normal EE from the control. A5, longitudinal section of an aberrant EE from the control. A.4 and A.6, longitudinal sections of aberrant EEs from line 35S:*WOX2i.4*. Note the vacuolated cells in the embryonal mass and the lack of distinct protoderm, owing to aberrant cell division of the cells in the outer cell layer of the embryonal mass denoted by arrows ⇧. Bar, 50 μm. **b** Frequency of normal EEs in the control and lines XVE-*WOX2i.12*, 35S:*WOX2i.2,* 35S:*WOX2i.3* and35S:*WOX2i.4* [non-induced (−), induced (+) with *β*-estradiol]. More than 100 EEs were analyzed from each line (Additional file 6: Table S3). Data are presented as means ± SE of three biological replicates. Asterisks indicate significant differences in the frequency of normal embryos between the control (−) and the transgenic lines (one tail *t*-test, *p* < 0.05). **c** Boxplot presenting the average length of about 50 suspensor cells from EEs in the control and line 35S:*WOX2i.4.* Box plot shows the median (solid line), the 25th and 75th percentiles (boxes), and the 5th and 95th percentiles (error bars). The presented data are based on about 20 embryos*.* Asterisk indicates significant difference in the length of suspensor cells between the control and the transgenic line (ANOVA, *p* < 0.05)

**Fig. 4:**
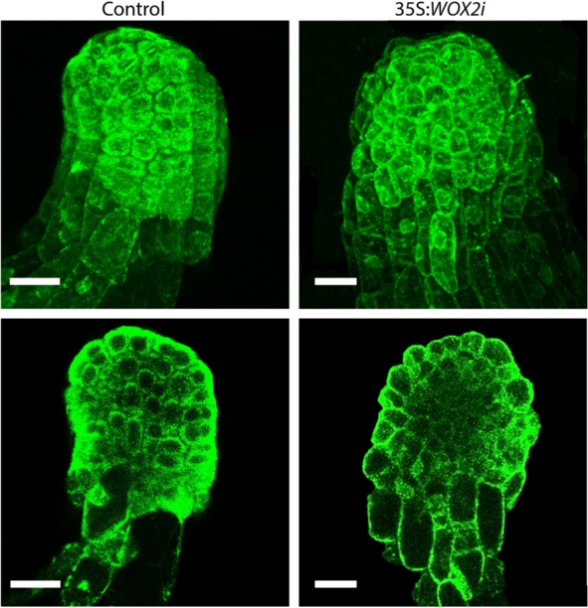
*PaWOX2* is important for correct differentiation of the protoderm. Images of LE1s in the control and line 35S:*WOX2i.4* were acquired by laser scanning confocal microscopy. Cells are visible due to their intrinsic autofluorescence, when excited with strong intensity argon laser line at 488 nm. Images are pseudocolored green. Upper tier, maximum projection of a Z-stack (30 sequential stack images, 2 μm each optical section); Bottom tier, single optical layer images of the embryos shown in the upper tier. Bar, 50 μm

A typical Norway spruce EE has a polarized structure with a distinct border between the compact globular embryonal mass in the apical part and elongated suspensor cells in the basal part (Fig. 3a.1). The embryonal mass consists of densely cytoplasmic cells delineated by a distinct protoderm with a smooth surface (Fig. 3a.3). Approximately 80 % of EEs from the control and 50 % of EEs from the 35S:*WOX2i* lines had normal morphology (Fig. 3b, Additional file 6: Table S3). The aberrant EEs in the control were characterized by a successive transition from small meristematic cells in the embryonal mass to elongated cells in the suspensor (Fig. 3a.5). The aberrant EEs in the 35S:*WOX2i* lines failed to establish distinct embryonal mass and suspensor domains. In these embryos the border between the embryonal mass and the suspensor was severely disturbed (Fig. 3a.4 and 6). In about one-third of the EEs in 35S:*WOX2i* lines the embryonal mass lacked a smooth surface and vacuolated cells were formed both from the outer cell layer and within the embryonal mass (Fig. 3a.2 and 6, Additional file 7: Table S4). This phenotype was rarely found in the control. Furthermore, the length of the suspensor cells in EEs of 35S:*WOX2i* lines were significantly reduced (Fig. 3c). Normal and aberrant LE1s were examined by confocal laser scanning microscopy (Fig. 4). The protoderm covering the embryonal mass on LE1s from the control was arranged in a structured way (Fig. 4). Protodermal cells were characterized by a rectangular cell-shape. In contrast, the embryonal mass in aberrant LE1s from the 35S:*WOX2i* lines lacked a distinct protoderm and a smooth surface. The aberrant morphology could also be observed in LE2s (data not shown).

**Fig. 5:**
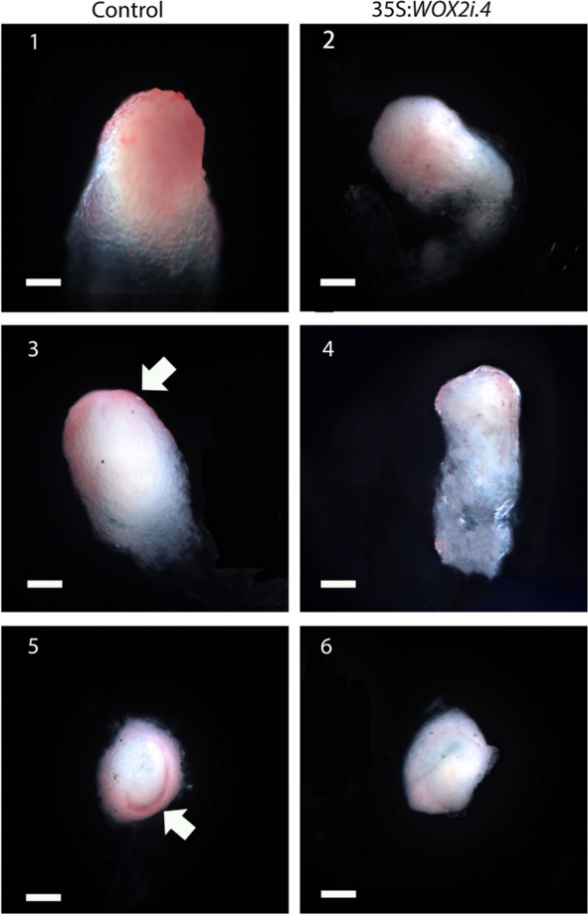
*PaWOX2* is important for the formation of a cuticularized layer. Freshly isolated LE1s from the control (1, 3 and 5) and line 35S:*WOX2i.4* (2, 4 and 6) were stained with Oil Red. 1 and 2, intact embryos; 3 and 4, longitudinal hand-sectioned embryos; 5 and 6, transverse hand-sectioned embryos. Note the strong positive staining on the surface of LE1s from the control, denoted by arrowheads. Bar, 50 μm

We used Oil Red staining to detect presence of the cuticularized layer in LE1s [28]. The staining was strong on surface of the embryonal mass in LE1s from the control, but weak and patchy on surface of LE1s from the 35S:*WOX2i* line (Fig. 5). This staining pattern indicates that the cuticularized layer is poorly developed around the embryonal mass in LE1s from the 35S:*WOX2i* line.

### PaWOX2 is not regulating cell division at the transcriptional level

Previously, we showed that *PaWOX8/9*, which specifies the cell division plane orientation in the basal part of the embryonal mass, regulates cell division at the transcriptional level [17]. In order to elucidate whether *PaWOX2* exerts a similar function, we compared the mRNA abundance of nine cell-cycle-regulating genes in EEs from the control and from line 35S:*WOX2i.4* by qRT-PCR. No significant differences in mRNA level of the tested cell-cycle-regulating genes could be observed between control and the RNAi line (Additional file 8: Figure S4). The previous suggests that *PaWOX2* and *PaWOX8/9* regulate the expression of different targets during embryo development.

### PaWOX2 is important for the development of mature embryos

**Fig. 6:**
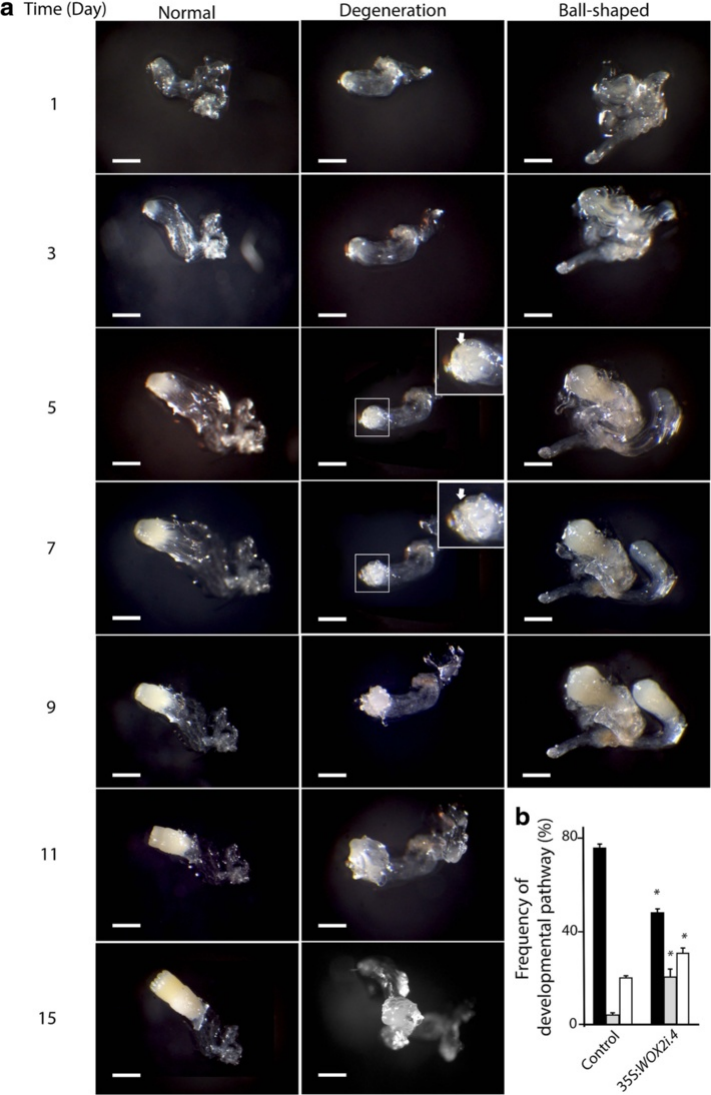
Developmental pathways of early embryos (EEs) in the control and line 35S:*WOX2i*.4. **a** The developmental pathway of EEs was followed by time-lapse tracking analysis during 15 days. EEs from the control cultures and line 35S:*WOX2i.4* were isolated after one week on maturation medium and transferred to fresh maturation medium. Representative images are shown for days 1, 3, 5, 7, 9, 11 and 15. The embryos followed three developmental pathways: i) normal development; ii) degeneration, in which the cells on the surface layer of the embryonal mass became vacuolated after five days, followed by initiation of new embryogenic tissue after nine days (the areas in the white squares are presented in higher magnification in the inserts; ⇧ arrows denote vacuolated surface layer); iii) development of ball-shaped embryos. The ball-shaped embryos did not develop further after 9 days. Bar, 100 μm. **b** Frequency of EEs that developed normally (black bars), went through the degeneration pathway (grey bars) or developed into ball-shaped embryos (white bars). The presented frequencies are based on the development of 80–90 embryos (Additional file 9: Table S5). Data are presented as means ± SE of three biological replicates. Asterisks indicate significant differences in the frequency of embryos in each developmental pathway between the control and the transgenic line (one tail *t*-test, *p* < 0.05)

To gain information about how the aberrations observed during early embryogenesis in *PaWOX2* RNAi lines affect further development of these embryos, we performed time-lapse tracking analysis during maturation. Individual EEs were selected from the control and *PaWOX2* RNAi lines. Three developmental pathways were observed: i) normal embryo maturation (Fig. 6a, Normal), ii) embryo degeneration, in which the cells on the surface layer of the embryonal mass became vacuolated, and new embryogenic tissue was initiated from the degenerated embryos (Fig. 6a, Degeneration), and iii) development of ball-shaped embryos, in which the embryos lacked differentiated cotyledons (Fig. 6a, Ball-shaped). Most of the EEs from the control developed into normal mature embryos (76 %), and only 4 % followed the degeneration pathway (Fig. 6b, Additional file 9: Table S5). About 50 % of the EEs from 35S:*WOX2i* lines developed normally. The frequency of EEs which developed into ball-shaped embryos was significantly higher in the 35S:*WOX2i* lines than in the control. However, an increased frequency of ball-shaped embryos was also observed in the transformed control (Additional file 9: Table S5), indicating that the formation of ball-shaped embryos is an artifact of the transgenic selection process. An increase in the frequency of ball-shaped embryos in transgenic lines of Norway spruce has been reported before [29]. Interestingly, the frequency of EEs that degenerated (on average 20 %) was significantly higher in the 35S:*WOX2i* lines, suggesting that this pathway is caused by down-regulation of *PaWOX2.*

**Fig. 7:**
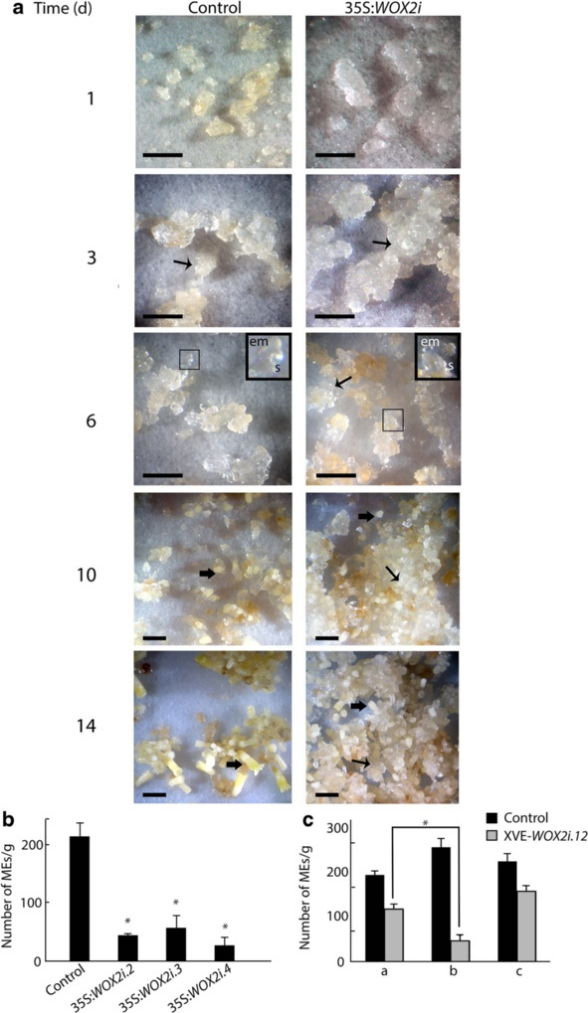
*PaWOX2* inhibits proliferation and promotes maturation. Embryogenic tissue from the control and three 35S:*WOX2i* lines were re-suspended in liquid medium after pre-maturation treatment and thereafter plated out as a thin layer on solidified maturation medium. Data are presented as means ± SE of three biological replicates. **a** The maturation process was examined for 14 days on maturation medium. The areas in the dark squares are presented in higher magnification in the inserts. Examples of proliferating tissues and MEs are denoted by arrows (↑, proliferating tissue. ⬆, ME). In the control culture: day 1 and 3, note the low proliferation of embryogenic tissue; day 6, note that LE1s have developed; day 10 and 14, note that MEs have developed. In the 35:*WOX2*i line: day 1 and 3, note the high proliferation of embryogenic tissue compared to the control; day 6, note that the embryogenic tissue continued to proliferate and LE1s had developed; day 10 to 14, note that the embryogenic tissue continued to proliferate and only a few MEs developed. em, embryonal mass; s, suspensor. Bar, 5 mm. **b** Number of MEs/gram tissue from the control and three 35S:*WOX2i* lines after seven weeks on maturation medium (Additional file 10: Table S6). Asterisks indicate significant differences in the number of MEs between the control and the transgenic lines (one tail *t*-test, *p* < 0.05). **c** Number of MEs/gram tissue formed in the control and line XVE-*WOX2i.12* after seven weeks on maturation medium (Additional file 11: Table S7). **a**: Non-induced cultures; **b**: Early-induced cultures were treated with *β*-estradiol from the first week on maturation medium; **c**: Late-induced cultures were treated with *β*-estradiol from the third week on maturation medium. Asterisk indicates significant difference in the number of MEs in line XVE-*WOX2i.12*, between non-induced (**a**), and induced from the first week on maturation medium (**b**) (one tail *t*-test, *p* < 0.05)

In order to examine the importance of *PaWOX2* for embryo maturation, cultures from control and 35:*WOX2*i lines were plated out as a thin layer on maturation medium. Control cultures ceased to proliferate during the first three days. EEs and LE1s were formed after six days and developed into MEs within two weeks (Fig. 7a). In contrast, in 35S:*WOX2i* lines, the proliferation of the embryogenic tissue did not decline on maturation medium. Although some LE1s had developed after six days (Fig. 7a), only a few LE1s developed into MEs. Consequently, the yield of MEs was significantly lower in 35S:*WOX2i* lines than in the control (Fig. 7b, Additional file 10: Table S6). However, the MEs formed had a normal morphology and 76 % germinated as compared to 80 % of MEs in the control line (data not shown).

For elucidating when during embryo development *PaWOX2* is important, we analyzed development of embryos of the inducible XVE-*WOX2i* lines. Initially 12 XVE-*WOX2i* lines treated with *β*-estradiol were screened for their potential to develop MEs. In 8 out of these 12 lines, the cultures continued to proliferate on maturation medium and the yield of MEs was low. Cell line XVE-*WOX2i.12* was choosen for further studies. The frequency of EEs with normal morphology was significantly lower in line XVE-*WOX2i.12 *induced with *β*-estradiol than in the control (Fig. 3b). For examining the role of *PaWOX2* in the formation of MEs, line XVE-*WOX2i.12* was induced with *β*-estradiol either from the first or from the third week on maturation medium. When down-regulation of *PaWOX2* was induced from the first week on maturation medium, the yield of MEs was significantly decreased (Fig. 7c, Additional file 11: Table S7). However, the yield of MEs was not affected when down-regulation of *PaWOX2* was induced at the third week on maturation medium, when LE2s had formed. These results suggest that the transient high expression of *PaWOX2* during the formation of EEs and LE1s is important for further development of the embryos.

## Discussion

In this study we show that the mRNA level of *PaWOX2* in somatic embryos is transiently up-regulated during early and late embryogeny. Results obtained by qRT-PCR analyses, were in good agreement with results obtained by *in situ* mRNA hybridization. The expression of *PaWOX2* in EEs and LE1s was detected in the embryonal mass and in suspensor cells that are in proximity to the embryonal mass. *In situ* mRNA analyses were also conducted on zygotic embryos. Owing to the difficulty to obtain high quality sections of the zygotic embryos we could not localize the signal to specific cell-types. However, we could detect signal in late embryos but not in maturing embryos. A comparable expression pattern for *PaWOX2* in somatic and zygotic embryos of Norway spruce has been reported by Palovaara, et al. [19]. Taken together, these results indicate that *PaWOX2* is expressed in a similar way in somatic and zygotic embryos during embryo development.

Hybridization signals could be detected in the region of the mega gametophyte that resides in front of the growing embryo. In Scots pine (*Pinus sylvestris*) it has been shown that a cone-shaped zone is formed in the mega gametophyte in front of the dominant embryo [30]. The cells in this zone are degenerating to make room for the actively growing embryo. We assume that the hybridization signals detected in our analyses correspond to the region in the mega gametophyte where the cells are committed to undergo cell death. However, at present we do not know why these cells are *PaWOX2*-positive.

To investigate the function of *PaWOX2*, embryogenic RNAi lines were established to down-regulate mRNA level of *PaWOX2*. EEs and LEs in35S:*WOX2i* lines showed aberrant morphology characterized by a severe disturbance of development in the border between the embryonal mass and the suspensor, decrease in the length of the suspensor cells and lack of the protoderm. Vacuolated cells formed in the outer cell layer of the embryonal mass and new embryogenic tissue was initiated from the embryonal mass. The previous result suggests that *PaWOX2* has a role in the regulation of early embryo development, both in the embryonal mass and the suspensor.

Normal EEs and LEs have a distinct border between the compact globular embryonal mass in the apical part and elongated suspensor cells in the basal part. In contrast, the border between the embryonal mass and the suspensor in embryos from 35S:*WOX2i* lines is severely disturbed. In Arabidopsis, *AtWOX2* shares redundant functions with *AtWOX8* in regulating embryonic apical patterning [15, 31]. Previously, we showed that *PaWOX8/9* is crucial for embryo patterning by regulating the orientation of the cell division plane at the basal part of the embryonal mass during early and late embryo development in Norway spruce. Expression level of cell-cycle-regulating genes, such as *E2F* is reduced in *PaWOX8/9* RNAi lines [17]. However, we could not detect any changes in mRNA levels of the tested cell-cycle-regulating genes in EEs from 35S:*WOX2*i lines. The previous suggests that *PaWOX2* may not control cell division at the transcriptional level as *PaWOX8/9* does, and that *PaWOX2* and *PaWOX8/9* might be involved in early embryo patterning through distinct pathways.

In Norway spruce, the most basally situated cells in the embryonal mass undergo asymmetric divisions to form the suspensor cells. Thereby, several files of suspensor cells are continuously formed. The suspensor cells do not divide but they elongate and become highly vacuolated [32]. In contrast, many suspensor cells in embryos from 35:*WOX2*i lines fail to elongate. The defect in the suspensor cells is consistent with the expression pattern of *PaWOX2. PaWOX2* is expressed in the embryonal mass and in suspensor cells that are in proximity to the embryonal mass. In Arabidopsis, expression of *AtWOX2* is limited to the upper tier [3] and no aberrant phenotype has been reported for the basal lineage of the embryos from the *wox2* mutant. Taken together, *PaWOX2* is functioning both in the embryonal mass and the suspensor while *AtWOX2* is only functioning in the apical part of the embryo proper.

In addition to the apical-basal polarity along the shoot-root axis, the basic body plan of plant embryos also shows a radial organization of primary tissue players: epidermis (derived from the protoderm), cortex and endodermis (derived from the ground tissue), pericycle and vascular tissues (derived from the procambium). Protoderm differentiation, which is the earliest event of radial pattern formation in plant embryogenesis [5, 33], is essential for normal patterning during early embryo development in both gymnosperms and angiosperms [9, 10, 34, 35]. In Norway spruce, normal EEs and LE1s have a compact globular embryonal mass covered by the protoderm. In contrast, the embryonal mass in EEs and LE1s from 35S:*WOX2i* lines with reduced *PaWOX2* mRNA level, lack a distinct protoderm. In a similar way, embryos from the Arabidopsis *wox2* mutant fail to correctly separate the protodermal layer by periclinal divisions in the upper part of the embryo [16]. We have previously shown that differentiation of the outer cell layer in the embryonal mass is also regulated by *PICEA ABIES HOMEOBOX1* (*PaHB1*), which encodes a protein highly similar to those from the *HD-GL2* angiosperm counterparts [9]. The *GL2*-type homeobox genes, e.g. *ARABIDOPSIS THALIANA MERISTEM LAYER 1* (*ATML1*) from Arabidopsis and *OVULE39* (*O39*) from *Phalaenopsis*, are considered to be a specialized subgroup of plant homeobox genes that exert a specific role in protoderm development [36]. This implies that the determination of the protoderm is regulated in a similar way in seed plants.

The epidermal cells secrete lipids and waxes to their outer cell wall. The previous results in the formation of a cuticle layer early during embryo development, after differentiation of the protoderm [7, 37]. The protoderm in normal LE1s is stained with Oil Red suggesting that the protoderm is covered by a cuticularized layer [28]. In contrast, LE1s from 35S:*WOX2*i lines either lack a cuticularized layer or the layer is thin and patchy, indicating that the function of some cells in the outer cell layer is disturbed.

Approximately one-third of the *wox2* Arabidopsis mutant embryos fail to correctly separate the protoderm layer by periclinal divisions in one or more cells of the prospective shoot region [16]. Intriguingly, at a later stage, most of these mutants (92 %) recover and give rise to fertile plants [3, 16]. In our study, about one-third of the EEs from line 35S:*WOX2i.4* showed an aberrant phenotype that is unique for 35S:*WOX2i* lines. Furthermore, the yield of MEs in the 35S:*WOX2i* lines is significantly reduced compared to the control. By using an inducible promoter we could show that down regulation of *PaWOX2* when LEs had already developed has no effect on further development of the embryos. Therefore, the transient high expression of *PaWOX2* during development of EEs and LEs is crucial for further development of LEs. The low recovering rate implies a more essential role for *PaWOX2* during early embryo development in Norway spruce compared to that of *AtWOX2* in Arabidopsis. However, an alternative scenario could be based on the previously suggested functional redundancy of *AtWOX1*, *AtWOX3* and *AtWOX*5 with *AtWOX2* [16]. It has been shown that the redundant functions of *AtWOX1*, *AtWOX3* and *AtWOX*5 become essential for normal development of the apical lineage in the absence of *AtWOX2* activity [16]. *WOX1* homologs have not been found in Norway spruce [13]. More research is required before we know if *WOX* genes in conifers exert redundant functions.

## Conclusions

*PaWOX2* is transiently expressed in the embryonal mass and in the upper part of the suspensor in early and early late embryos. Our results suggest that *PaWOX2* is required for the establishment of embryonal mass and suspensor domains, expansion of suspensor cells and proper protoderm formation during early embryo development. EEs that fail to form a protoderm cannot develop further and degenerate. Taken together, our results suggest that *WOX2* exerts a conserved role in protoderm development in both gymnosperms and angiosperm, whereas its function in the expansion of suspensor cells may be restricted to the gymnosperm lineage.

## Additional files

Additional file 1: Table S1. Primer sequences used for qRT-PCR and *in situ* hybridization probe (*) of *PaWOX2* and corresponding reference genes. (DOCX 12 kb) Additional file 2: Figure S1. Schematic representation of *PaWOX2* coding sequence. (DOCX 44 kb) Additional file 3: Table S2. Primer sequences used for vector construction of *PaWOX2* interference. (DOCX 11 kb) Additional file 4: Figure S2. Quantitative RT-PCR analysis of the relative mRNA level of *PaWOX2* in early late embryos (LE1s). (DOCX 683 kb) Additional file 5: Figure S3. Expression pattern of *PaWOX2* in zygotic embryos. (DOCX 608 kb) Additional file 6: Table S3. Frequency of early embryos (EEs) with normal morphology in the control and *PaWOX2* RNAi lines. (DOCX 13 kb) Additional file 7: Table S4. Frequency of early embryos (EEs) lacking a smooth surface on the embryonal mass in the control and *PaWOX2* RNAi lines. (DOCX 12 kb) Additional file 8: Figure S4. Quantitative real-time PCR analysis of the mRNA level of cell-cycle-regulating genes in control and line 35S:*WOX2i.4*. (DOCX 160 kb) Additional file 9: Table S5. Developmental pathways of early embryos (EEs) in control and 35S:*WOX2i* lines. (DOCX 15 kb) Additional file 10: Table S6. Formation of mature embryos (MEs) in the control and 35S:*WOX2i.*lines. (DOCX 12 kb) Additional file 11: Table S7. Development of mature embryos (MEs) in the control and line XVE-*WOX2i.12*. (DOCX 12 kb)
