# Supplementary material for: WUSCHEL-RELATED HOMEOBOX 2 is important for protoderm and suspensor development in the gymnosperm Norway spruce
Source: BMC Plant Biol. 2016 Jan 19;16:19. doi: 10.1186/s12870-016-0706-7 (PMC4719685; doi:10.1186/s12870-016-0706-7)
Supplement: Additional file 1: Table S1. — Primer sequences used for qRT-PCR and in situ hybridization probe (*) of PaWOX2 and corresponding reference genes. (DOCX 12 kb) [file 12870_2016_706_MOESM1_ESM.docx]

**Additional file 5**

**Table S1.** Primer sequences used for qRT-PCR and *in situ* hybridization probe (*) of *PaWOX2* and corresponding reference genes.

| Gene | Forward primer | Reverse primer |
| --- | --- | --- |
| *PaWOX2* | CCAGATGGAATCCAACGAAAGAAC | TGAGGAGGGAGAACTTCTGTATGTG |
| **PaWOX2* | taatacgactcactataggg  CCTTCCTTCTCAGGCGCATC | aattaaccctcactaaaggga  CCCGCCTGAGCTTGCATCGTT |
| *PaEF1* | CACCTTGGGAGTGAAGCAAATG | GGGAGTAGTGGCATCCATCTTG |
| *PaCDC2* | TCCGACGGGTGCAGAGAA | GCTCCATTCAGCCTGATTCAA |
| *PaPHOS* | AATGCAGTTGAAGCCATTCC | CCAGTGCCGAAACTCTCTTC |
