# Supplementary material for: WUSCHEL-RELATED HOMEOBOX 2 is important for protoderm and suspensor development in the gymnosperm Norway spruce
Source: BMC Plant Biol. 2016 Jan 19;16:19. doi: 10.1186/s12870-016-0706-7 (PMC4719685; doi:10.1186/s12870-016-0706-7)
Supplement: Additional file 2: Figure S1. — Schematic representation of PaWOX2 coding sequence. (DOCX 44 kb) [file 12870_2016_706_MOESM2_ESM.docx]

# Additional file 1

**
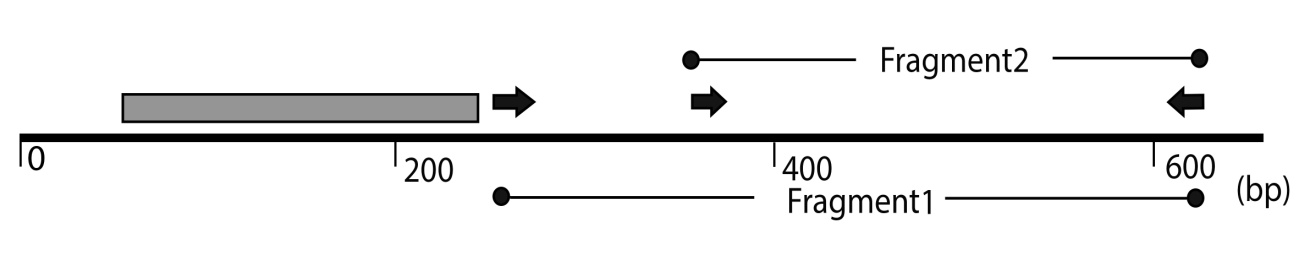
**

**Figure S1. Schematic representation of *PaWOX2* coding sequence.** The gray box indicates the homeodomain. The black arrows indicate the location of the primers used for the preparation of the RNAi construct. Primer sequences are presented in table S1.
