# Supplementary material for: WUSCHEL-RELATED HOMEOBOX 2 is important for protoderm and suspensor development in the gymnosperm Norway spruce
Source: BMC Plant Biol. 2016 Jan 19;16:19. doi: 10.1186/s12870-016-0706-7 (PMC4719685; doi:10.1186/s12870-016-0706-7)
Supplement: Additional file 3: Table S2. — Primer sequences used for vector construction of PaWOX2 interference. (DOCX 11 kb) [file 12870_2016_706_MOESM3_ESM.docx]

**Additional file 6**

**Table S2.** Primer sequences used for vector construction of *PaWOX2* RNA interference.

The underlined sequences show enzyme digestion sites. Sequence in lowercase designates the sequence required by the S/TOPO cloning vector.

| Fragment | Forward primer | Reverse primer |
| --- | --- | --- |
| 1 | caccTTGCCAGGATGCTGAGGG | GAATTCGGATCCG  GGGTATTCAGCAATCAGTTTCT |
| 2 | GAATTCGGATCCG  AAAGGTTGGTTCCACGCTAAC | TTGGCGCGCCTTGCCAGGATGCTGAGGG |
