# Supplementary material for: WUSCHEL-RELATED HOMEOBOX 2 is important for protoderm and suspensor development in the gymnosperm Norway spruce
Source: BMC Plant Biol. 2016 Jan 19;16:19. doi: 10.1186/s12870-016-0706-7 (PMC4719685; doi:10.1186/s12870-016-0706-7)
Supplement: Additional file 4: Figure S2. — Quantitative RT-PCR analysis of the relative mRNA level of PaWOX2 in early late embryos (LE1s). (DOCX 683 kb) [file 12870_2016_706_MOESM4_ESM.docx]

**Additional file 2**

**
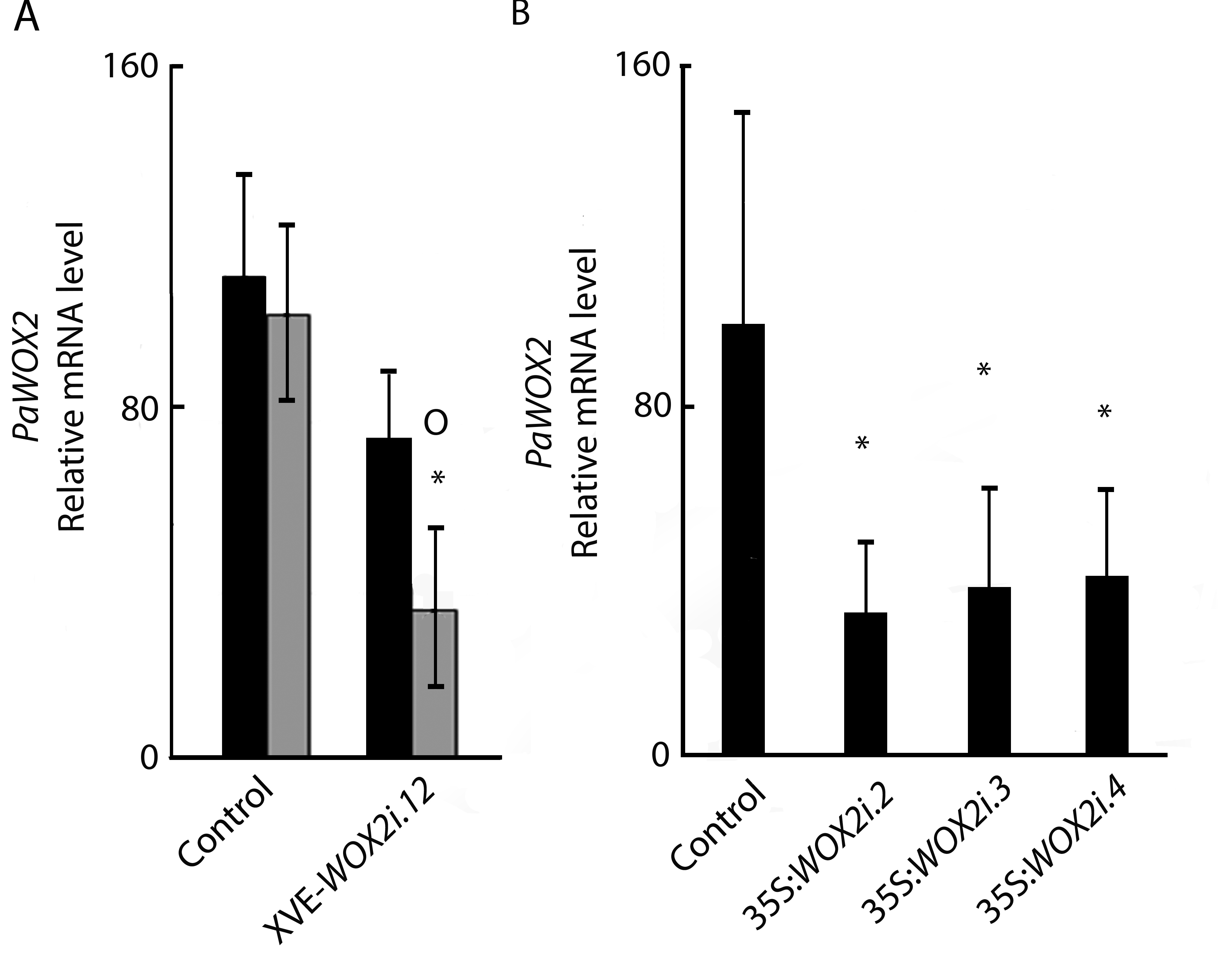
**

**Figure S2. Quantitative RT-PCR analysis of the relative mRNA level of *PaWOX2* in early late embryos (LE1s).** LE1s were sampled after two weeks on maturation medium from the control, 35S:*WOX2i* and XVE-*WOX2i* lines. The mRNA level of *PaWOX2* was normalized against three reference genes: *CELL DIVISION CONTROL2 (PaCDC2), ELONGATION FACTOR 1 (PaEF1)* and *PHOSPHOGLUCOMUTASE (PaPHOS)*. The presented data are means ± SE of three biological replicates. A) Relative mRNA level of *PaWOX2* in line XVE-*WOX2i.12*. Relative mRNA level of *PaWOX2* in control and line XVE-*WOX2i*.*12*, non-induced (black bars) or induced for 48h with *β*-estradiol (grey bars). Asterisks indicate significant differences between the control and the line XVE-*PaWOX2i.12* (ANOVA , p<0.05); circles indicate significant difference between the non-induced and induced cultures (ANOVA, p<0.05). B) Relative mRNA level of *PaWOX2* in 35S:*WOX2i* (2-4). Asterisks indicate significant differences between the control and the 35S:*PaWOX2i* lines (two tail t-test, p<0.05).
