# Supplementary material for: WUSCHEL-RELATED HOMEOBOX 2 is important for protoderm and suspensor development in the gymnosperm Norway spruce
Source: BMC Plant Biol. 2016 Jan 19;16:19. doi: 10.1186/s12870-016-0706-7 (PMC4719685; doi:10.1186/s12870-016-0706-7)
Supplement: Additional file 5: Figure S3. — Expression pattern of PaWOX2 in zygotic embryos. (DOCX 608 kb) [file 12870_2016_706_MOESM5_ESM.docx]

**Additional file 3**


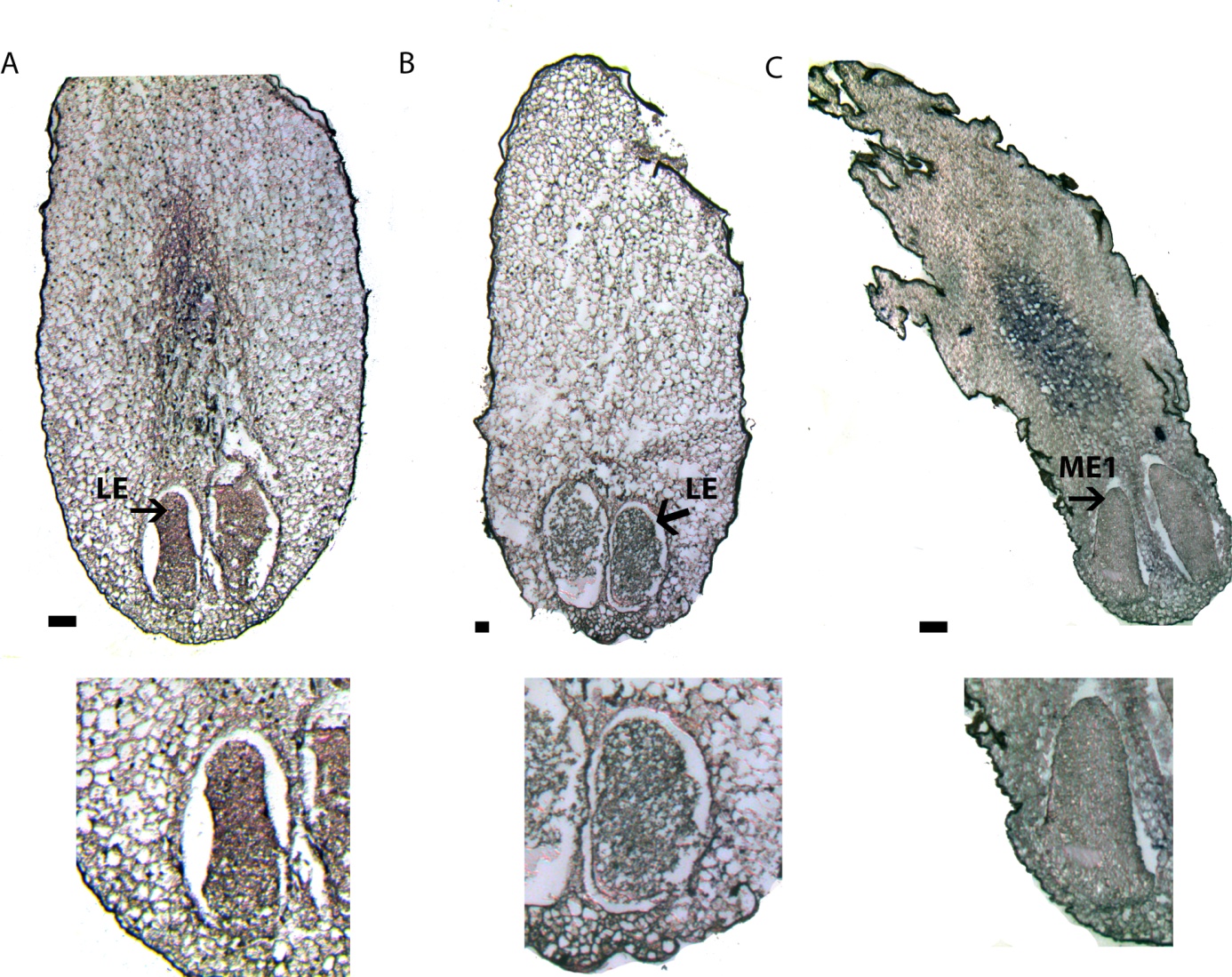


**Figure S3 Expression pattern of *PaWOX2* in zygotic embryos.**

Ovules from cones collected in the end of June were fixed and embedded for *in situ* hybridization*.* Hybridization signals appear as dark blue in bright field microscopy. The embryos are presented in higher magnification below the photos of the whole ovules. (A and B) (A) Anti-sense probe. Note signal in the embryo (arrow) and in the region of the mega gametophyte residing in front of the growing embryo. (B) Sense probe (negative control). Note the lack of signals in the embryo (arrow). (C) Section of an ovule containing maturing embryos. Note the lack of signal in the embryos but the strong signal in the region of the mega gametophyte residing in front of the growing embryo. Bar = 100 µm.
