# Supplementary material for: WUSCHEL-RELATED HOMEOBOX 2 is important for protoderm and suspensor development in the gymnosperm Norway spruce
Source: BMC Plant Biol. 2016 Jan 19;16:19. doi: 10.1186/s12870-016-0706-7 (PMC4719685; doi:10.1186/s12870-016-0706-7)
Supplement: Additional file 6: Table S3. — Frequency of early embryos (EEs) with normal morphology in the control and PaWOX2 RNAi lines. (DOCX 13 kb) [file 12870_2016_706_MOESM6_ESM.docx]

**Additional file 7**

**Table S3.** Frequency of early embryos (EEs) with normal morphology in the control and *PaWOX2* RNAi lines.

EEs from the control and lines 35S:*WOX2i.2*, 35S:*WOX2i.3,* 35S:*WOX2i.4*, XVE-*WOX2i.12* were, after one week on maturation medium, classified as normal or aberrant. Presented data shows the frequency of normal EEs. ‘+’ indicate that the maturation medium contained 10 µM *β*-estradiol. Each analysis was performed with three biological replicates (a, b, c).

| Line | Replicate | Total number  of EEs | Frequency of  EEs with normal morphology ( %) |
| --- | --- | --- | --- |
| Control | a | 221 | 82 |
|  | b | 127 | 85 |
|  | c | 136 | 83 |
|  | a+ | 121 | 79 |
|  | b+ | 96 | 78 |
|  | c+ | 128 | 85 |
| 35S:*WOX2i.2* | a | 132 | 52 |
|  | b | 203 | 62 |
|  | c | 163 | 62 |
| 35S:*WOX2i.3* | a | 134 | 61 |
|  | b | 161 | 64 |
|  | c | 152 | 64 |
| 35S:*WOX2i.4* | a | 93 | 45 |
|  | b | 138 | 56 |
|  | c | 82 | 40 |
| XVE-*WOX2i.12* | a | 87 | 76 |
|  | b | 162 | 81 |
|  | c | 117 | 67 |
|  | a+ | 104 | 62 |
|  | b+ | 176 | 64 |
|  | c+ | 165 | 58 |
