# Supplementary material for: WUSCHEL-RELATED HOMEOBOX 2 is important for protoderm and suspensor development in the gymnosperm Norway spruce
Source: BMC Plant Biol. 2016 Jan 19;16:19. doi: 10.1186/s12870-016-0706-7 (PMC4719685; doi:10.1186/s12870-016-0706-7)
Supplement: Additional file 7: Table S4. — Frequency of early embryos (EEs) lacking a smooth surface on the embryonal mass in the control and PaWOX2 RNAi lines. (DOCX 12 kb) [file 12870_2016_706_MOESM7_ESM.docx]

**Additional file 8**

**Table S4.** Frequency of early embryos (EEs) lacking a smooth surface on the embryonal mass in the control and 35S: *WOX2i* lines.

The frequency of EEs with vacuolated cells in the outer cell layer of the embryonal mass in the control and lines 35S:*WOX2i.2*, 35S:*WOX2i.3,* 35S:*WOX2i.4* were analyzed after one week on maturation medium. Each analysis was performed with three biological replicates (a, b, c).

| Line | Replicate | Total number  of EEs | Frequency of aberrant  EEs ( %) |
| --- | --- | --- | --- |
| Control | a | 177 | 1 |
|  | b | 128 | 0 |
|  | c | 165 | 1 |
| 35S:*WOX2i.2* | a | 133 | 23 |
|  | b | 167 | 25 |
|  | c | 182 | 21 |
| 35S:*WOX2i.3* | a | 133 | 22 |
|  | b | 125 | 20 |
|  | c | 134 | 22 |
| 35S:*WOX2i.4* | a | 78 | 32 |
|  | b | 146 | 34 |
|  | c | 104 | 31 |
