# Supplementary material for: WUSCHEL-RELATED HOMEOBOX 2 is important for protoderm and suspensor development in the gymnosperm Norway spruce
Source: BMC Plant Biol. 2016 Jan 19;16:19. doi: 10.1186/s12870-016-0706-7 (PMC4719685; doi:10.1186/s12870-016-0706-7)
Supplement: Additional file 8: Figure S4. — Quantitative real-time PCR analysis of the mRNA level of cell-cycle-regulating genes in control and line 35S:WOX2i.4. (DOCX 160 kb) [file 12870_2016_706_MOESM8_ESM.docx]

**Additional file 4**


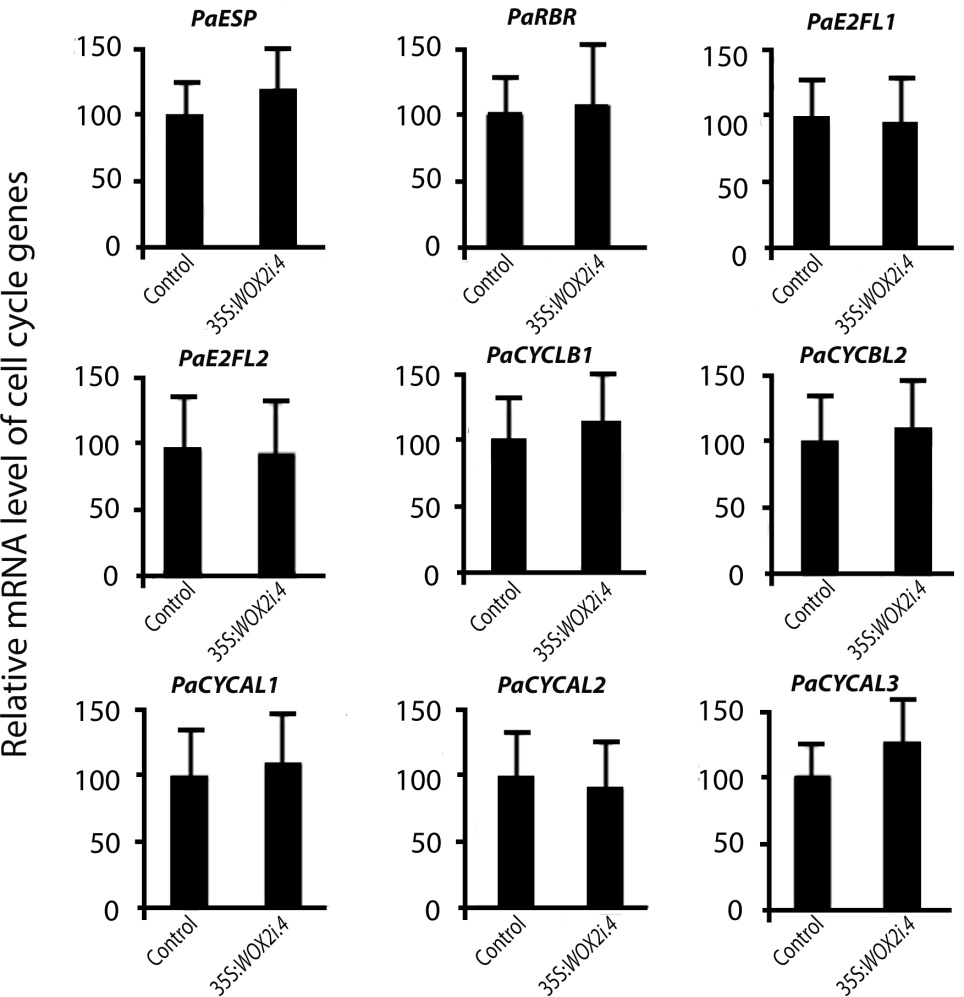


**Figure S4. Quantitative real-time PCR analysis of the relative mRNA level of cell-cycle-regulating genes in control and line 35S:*WOX2i.4*.** The transcript level of cell-cycle-regulating genes was analyzed in EEs in the control and line 35S:*WOX2i.4*. The transcript level of the following genes was analyzed: *PaESP, PaRBR, PaE2FL1, PaE2FL2* and *PaCYCBL1, PaCYCBL2, PaCYCAL1, PaCYCAL2* and *PaCYCAL3*. The mRNA levels are relative to the transcript level of each gene in EEs in the control and normalized against three reference genes: *PaCDC2, PaEF1* and *PaPHOS*. The presented mRNA levels are means ± SD of two biological replicates.
