# Supplementary material for: WUSCHEL-RELATED HOMEOBOX 2 is important for protoderm and suspensor development in the gymnosperm Norway spruce
Source: BMC Plant Biol. 2016 Jan 19;16:19. doi: 10.1186/s12870-016-0706-7 (PMC4719685; doi:10.1186/s12870-016-0706-7)
Supplement: Additional file 9: Table S5. — Developmental pathways of early embryos (EEs) in control and 35S:WOX2i lines. (DOCX 15 kb) [file 12870_2016_706_MOESM9_ESM.docx]

**Additional file 9**

**Table S5.** Developmental pathways of early embryos (EEs) in 35S:*WOX2i* lines.

The development of randomly selected EEs in the untransformed control (Control), transformed control (T-control, 2x35S:*GUS*) and lines *35S:WOX2i.2, 35S:WOX2i.3* and 35S:*WOX2i.4* was followed by time-laps tracking analyses during 15 days. EEs were sampled after one week on maturation medium and transferred to fresh maturation medium. Three developmental pathways were recorded (Fig. 5): i) Normal development, ii) Degeneration, in which the cells on the surface layer of the embryonal mass became vacuolated followed by initiation of embryogenic tissue, and iii) Ball-shaped, development of ball-shaped embryos. The experiment was repeated twice for the control and line 35S:*WOX2i.4.* The total number of tracked EEs is presented for each replicate (a, b, c).

| Line | Replicate | Total number of EEs | Developmental pathway (%) | | |
| --- | --- | --- | --- | --- | --- |
|  |  |  | Normal | Degeneration | Ball-shaped |
| Control | a | 77 | 73 | 5 | 22 |
|  | b | 82 | 78 | 2 | 20 |
|  | c | 77 | 77 | 4 | 19 |
| T-control | a | 92 | 64 | 1 | 35 |
| 35S:*WOX2i.2* | a | 85 | 46 | 16 | 38 |
| 35S:*WOX2i.3* | a | 77 | 53 | 17 | 30 |
| 35S:*WOX2i.4* | a | 82 | 48 | 26 | 26 |
|  | b | 94 | 51 | 14 | 35 |
|  | c | 94 | 47 | 22 | 31 |
