# Supplementary material for: WUSCHEL-RELATED HOMEOBOX 2 is important for protoderm and suspensor development in the gymnosperm Norway spruce
Source: BMC Plant Biol. 2016 Jan 19;16:19. doi: 10.1186/s12870-016-0706-7 (PMC4719685; doi:10.1186/s12870-016-0706-7)
Supplement: Additional file 10: Table S6. — Formation of mature embryos (MEs) in the control and 35S:WOX2i.lines. (DOCX 12 kb) [file 12870_2016_706_MOESM10_ESM.docx]

**Additional file 10**

**Table S6.** Formation of mature embryos (MEs) in the control and 35S:*WOX2i.* lines.

Number of MEs formed per gram tissue in the control and lines 35S:*WOX2i.2*, 35S:*WOX2i.3* and 35S:*WOX2i.4* after seven weeks on maturation medium. Each analysis was performed with three or five biological replicates (a, b, c, d, e).

| Replicate | | Line | | | | | | | |
| --- | --- | --- | --- | --- | --- | --- | --- | --- | --- |
|  |  | Control | | 35S:*WOX2i.2* | | 35S: *WOX2i.3* | | 35S: *WOX2i.4* | |
| a | 264 | | 40 | | 16 | | 0 | |  |
| b | 229 | | 41 | | 89 | | 0 | |  |
| c | 205 | | 50 | | 64 | | 30 | |  |
| d | 168 | | - | | - | | 45 | |  |
| e | 217 | | - | | - | | 54 | |  |
