# Supplementary material for: WUSCHEL-RELATED HOMEOBOX 2 is important for protoderm and suspensor development in the gymnosperm Norway spruce
Source: BMC Plant Biol. 2016 Jan 19;16:19. doi: 10.1186/s12870-016-0706-7 (PMC4719685; doi:10.1186/s12870-016-0706-7)
Supplement: Additional file 11: Table S7. — Development of mature embryos (MEs) in the control and line XVE-WOX2i.12. (DOCX 12 kb) [file 12870_2016_706_MOESM11_ESM.docx]

**Additional file 11**

**Table S7.** Development of mature embryos (MEs) in the control and line XVE-*WOX2i.12*.

Number of MEs formed per gram tissue in the control and line XVE-*WOX2i.12* after seven weeks on maturation medium. Non-induced, not treated with *β*-estradiol; Early-induced, treated with *β*-estradiol from the first week on maturation medium; Late-induced, treated with *β*-estradiol from the third week on maturation medium. Data from three biological replicates (a, b, c) are presented.

| Line | Replicate | Non-induced | Early-induced | Late-induced |
| --- | --- | --- | --- | --- |
| Control | a | 205 | 198 | - |
|  | b | 168 | 313 | 187 |
|  | c | 217 | 269 | 267 |
| XVE-*WOX2i.12* | a | 75 | 11 | - |
|  | b | 147 | 41 | 187 |
|  | c | 133 | 87 | 131 |
